# Development of a Syndromic Molecular Diagnostic Assay for Tick-Borne Pathogens Using Barcoded Magnetic Bead Technology

**DOI:** 10.1128/spectrum.04395-22

**Published:** 2023-05-11

**Authors:** Nazleeen Mohseni, Mariann Chang, Kathryn Garcia, Mina Weakley, Tram Do, Sheema Mir

**Affiliations:** a College of Veterinary Medicine, Western University of Health Sciences, Pomona, California, USA; University of California, Davis

**Keywords:** barcoded magnetic beads, high-throughput, tick-borne pathogens

## Abstract

Infectious disease diagnostics often depend on costly serological testing with poor sensitivity, low specificity, and long turnaround time. Here, we demonstrate proof of the principle for simultaneous detection of two tick-borne pathogens from a single test sample using barcoded magnetic bead technology on the BioCode 2500 system. Specific primer sets complementary to the conserved genes of Anaplasma phagocytophilum and Borrelia burgdorferi were used in PCR amplification of the target, followed by the hybridization of the resulting biotinylated PCR products with specific probes tethered to the barcoded magnetic beads for simultaneous detection, using a fluorophore with high quantum yield. The assay has an extremely high signal to background ratio, with a limit of detection (LOD) of 2.81 50% tissue culture infection dose (TCID_50_)/mL and 1 CFU/mL for A. phagocytophilum and B. burgdorferi, respectively. The observed LOD for gene blocks was 1.8 copies/reaction for both the pathogens. The assay demonstrated 100% positive and negative agreement on performance evaluation using patient specimens and blood samples spiked with 1 × LOD of pathogen stock. No cross-reactivity was observed with other related tick-borne pathogens and genomic DNA of human, cattle, and canine origin. The assay can be upgraded to a sensitive and cost-effective multiplex diagnostic approach that can simultaneously detect multiple clinically important tick-borne pathogens in a single sample with a short turnaround time.

**IMPORTANCE** The low pathogen load in the tick-borne disease test samples and the lack of highly sensitive multiplex diagnostic approaches have impacted diagnosis during clinical testing and limited surveillance studies to gauge prior insight about the prevalence of tick-borne infections in a geographical area. This article demonstrates proof of the principle for simultaneous detection of two important tick-borne pathogens from a single test sample using digital barcoded magnetic bead technology. Using a fluorophore of high quantum yield, the diagnostic approach showed high sensitivity and specificity. The LOD was 1.8 genome copies per reaction for both A. phagocytophilum and B. burgdorferi. The assay can be upgraded for the detection of all clinically important tick-borne pathogens from a single patient sample with high sensitivity and specificity. The assay can provide a diagnostic answer to the clinician in a short turnaround time to facilitate speedy therapeutic intervention to infected patients and implement public health measures to prevent community spread.

## INTRODUCTION

Ticks are the principal vectors for both animal and human blood-borne pathogens causing a variety of diseases, such as babesiosis, anaplasmosis, Lyme disease, Crimean Congo hemorrhagic fever, and Rocky Mountain spotted fever. These tick-borne diseases are seeing greater emergence due to the geographical expansion of ticks worldwide. The rapid geographical expansion and overpopulation of ticks is due to global warming, which supports tick growth (1). In the United States, the incidence and geographic range of blacklegged tick Ixodes scapularis in the east and Ixodes pacificus in the west, have dramatically increased in recent decades. Both have been shown to transmit many pathogens, including *Anaplasma* and *Borrelia* spp. (2). Inside ixodid ticks these pathogens are known to survive in salivary glands and midgut cells. Borrelia burgdorferi causes Lyme disease (LD), the most common tick-borne infection in North America (3). A direct link between B. burgdorferi and LD was established with its isolation from the blood of human LD patients (4). To date, 21 distinct species associated with LD have been identified (5). B. burgdorferi is an obligate host-dependent bacterium maintained in a natural enzootic cycle involving diverse small mammal reservoir species and *Ixodes* tick vectors (6). Human transmission occurs through the bite of an infected tick. Classic clinical symptoms include fever, headache, fatigue, and a hallmark early skin lesion called erythema migrans. If left untreated, late manifestations involve the joints, heart, and nervous system, causing significant and debilitating disease (7). A bite from an infected tick may also lead to coinfection with other tick-borne diseases such as anaplasmosis (8). Preventive measures are focused on encouraging people to use repellents, wear protective clothing, and check themselves for ticks (9, 10). The human health toll and socioeconomic impact of LD are staggering. The rapid and ongoing geographic spread of Ixodes scapularis and Ixodes pacificus ticks consistent with the increase in LD cases is well documented (11). The investigational vaccines were developed for human use in 1990s (ImuLyme, Pasteur-Merieux-Connaught, and LYMErix; SmithKline Beecham). The next-generation LD vaccines for humans are undergoing clinical trials (7). The prognosis of LD is excellent If diagnosed early and treated appropriately. After a recognized tick bite, antibiotics such as doxycycline are prescribed (12). There are currently no FDA-approved treatments for patients who have undergone a recommended course of antibiotics for LD but continue to have persistent symptoms. Diagnosis involves the culturing of B. burgdorferi from the test sample, followed by enzyme-linked immunosorbent assay (ELISA) and Western blot testing for both IgM and IgG antibodies (13). The CDC approved two-tiered testing that includes a screening test by an ELISA, followed by a specificity test by Western blotting. This testing approach is inaccurate in the early stages of disease due to its reliance on a host-antibody response that often takes 3 weeks or more to develop (14–16). While B. burgdorferi is recalcitrant to culturing under laboratory conditions, a clinically relevant PCR assay for the detection of LD from blood samples has not been established due to the insufficient sensitivity of conventional PCR methods and the extremely low levels of spirochetes found in the blood of infected patients (14–16).

Anaplasma phagocytophilum, a Gram-negative obligate intracellular rickettsial pathogen causes human granulocytic anaplasmosis (HGA). It is the third most common vector-borne diseases in the United States and cases have increased dramatically in recent decade (17). After infection in mammals, Anaplasma phagocytophilum colonizes in neutrophils and infects other cells of myeloid and nonmyeloid origin (18). Humans remain “dead-end” hosts after infection, and the molecular basis for severe disease remains elusive (19). A. phagocytophilum also infects other mammals such as horses, dogs, and sheep and causes a systemic disease with clinical symptoms similar to those of humans. Clinical symptoms include unexplained fever, myalgia, headache, malaise, thrombocytopenia, leukopenia, anemia, and mild-to-moderate hepatic injury within 3 weeks of exposure to *Ixodes* ticks (19). A. phagocytophilum uses several strategies that contribute to its disease pathogenesis, including its ability to alter neutrophil mobilization of phagocyte oxidase by disorganized assembly, superoxide dismutase activity, and reduced transcription of key phagocyte oxidase genes (20), and its ability to induce upregulated chemokine expression and neutrophil vesicle discharge leads to the recruitment of new host neutrophils to support the expansion of the pathogen population (21). Immunologic control of A. phagocytophilum is not fully understood; however, it includes both humoral and cellular responses. The inflammatory disease is most likely due to immunopathologic injury after gamma interferon (IFN-γ) activation (19). Diagnosis involves identification of the characteristic intragranulocytic inclusions on blood smear and testing for antibodies to A. phagocytophilum. CDC recommends the evidence of seroconversion or a 4-fold increase in titers between acute and convalescent serologic testing for confirming the diagnosis. The serologic tests have been shown to have poor sensitivity during the acute phases of infection, especially within 1 week of the onset of disease symptoms (22). The PCR-based diagnostic tests are highly recommended due to their higher sensitivity during the acute phase of illness. The real-time PCR-based assays can detect 5 to 10 copies of the A. phagocytophilum target DNA in the test sample (23).

Both anaplasmosis and Lyme borreliosis account for tens of thousands of reported cases of tick-borne disease every year (24). Given the nonspecific and undifferentiated nature of clinical symptoms, definitive diagnosis and treatment remain a challenge. The low pathogen load in the test sample and the lack of FDA-approved nucleic acid-based multiplex diagnostic approaches continue to keep these diseases underdiagnosed during the acute phase of illness (25), delaying the appropriate and timely preventive and therapeutic interventions. The PCR-based methods (26) have shown high specificity and sensitivity compared to the indirect fluorescent antibody test (IFAT), the enzyme-linked immunosorbent assay (ELISA), the complement fixation test (CFT), Western blots, and the immunochromatography test (ICT) (27, 28). To our knowledge none of the PCR-based assays have been approved by the FDA for clinical diagnosis. The need of the hour is to develop a high-throughput molecular diagnostic approach that can simultaneously test the presence of the two above-described important tick-borne pathogens in the patient sample and provide a highly informative diagnostic picture to the clinician (29). The multiplex assays are fast and cost-effective compared to single-target PCR for screening and detecting pathogens in test samples. Applied BioCode previously used barcoded magnetic bead (BMB) technology for the development of FDA-approved diagnostic assays that are currently used for clinical testing nationwide. For example, an FDA-approved 510k respiratory pathogen panel (510k RPP) developed by Applied BioCode (30) simultaneously detects multiple upper respiratory track pathogens, including viruses (adenovirus, coronavirus 229E, coronavirus HKU1, coronavirus NL63, coronavirus OC43, influenza A-H1, seasonal-H1N1, 2009-H3 subtype, influenza B, metapneumovirus (HMPV), parainfluenza virus type 1, parainfluenza virus type 2, parainfluenza virus type 3, parainfluenza virus type 4, respiratory syncytial virus (RSV), rhinovirus/enterovirus, and three bacterial targets (Bordetella pertussis, Chlamydophila pneumonia, Mycoplasma pneumoniae) from a single patient sample. Applied BioCode also used this technology for the development of an FDA-approved 510k gastrointestinal pathogen panel (510k GPP) for the detection of multiple pathogens that cause gastrointestinal infections in humans) (31).

Here, we report the development of a high-throughput multiplex PCR-based diagnostic assay using barcoded magnetic bead (BMB) technology that simultaneously detects A. phagocytophilum and B. burgdorferi from a single test sample with high sensitivity and specificity.

## RESULTS

### Technology overview.

**FIG 1:**
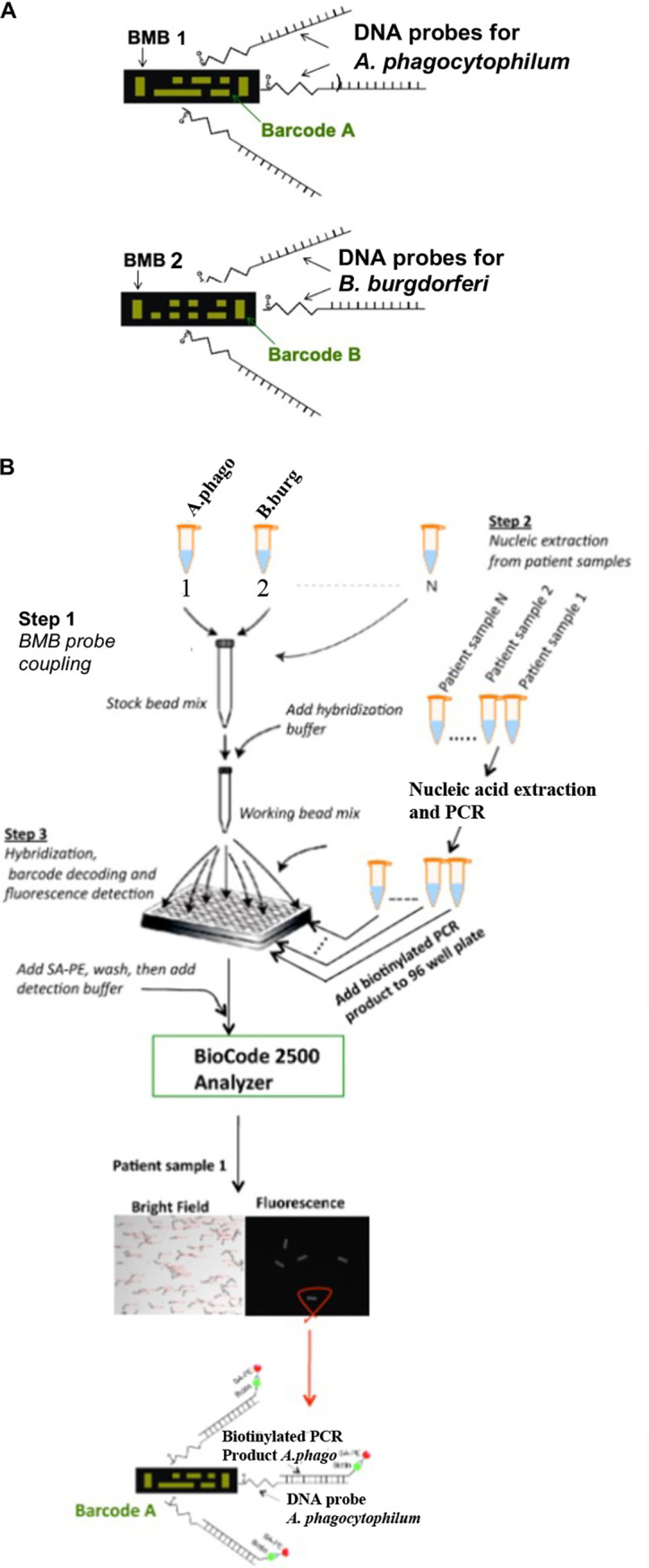
(A) Barcoded magnetic bead (BMB). An example of BMBs showing the barcode (green) on a transparent polymer bead. Shown are the two BMBs (BMB-1 and BMB-2) with barcodes A and B, respectively, chemically linked to the DNA probes specific for the identification of A. phagocytophilum or B. burgdorferi, respectively. (B) Assay workflow. Step 1, hybridization of DNA probes with BMBs; step 2, nucleic acid extraction from the test samples of interest, followed by PCR amplification of target amplicons; step 3, hybridization, fluorescence signal detection, barcode scanning, and decoding to reveal the sample identity.

Applied BioCode’s barcoded magnetic bead (BMB) technology uses a transparent magnetic polymer bead encapsulated with a digital barcode (Fig. 1A). These barcoded magnetic beads are manufactured using well-established and highly reproducible semiconductor processes. All beads are created equally, but their barcodes vary. Beads with 4,096 different barcodes are available. Each barcode is identified by the barcode identification system of the instrument. Each bead with a particular barcode is chemically linked to a DNA test probe (Fig. 1A). It must be noted that numerous copies of the same test probe are linked to a single bead to increase the sensitivity for target detection. The test probes are 20- to 27-nucleotide-long single-strand DNA segments complementary to the target gene of the pathogen of interest. The workflow for the development of the proposed assay includes the following steps.

**(i) Step 1: BMB coupling with the DNA probe.** Barcoded magnetic beads referred to as BMB-1 and BMB-2 with two different barcodes, shown as barcode A and barcode B (Fig. 1A), were linked with probes complementary to major surface protein and flagellin in Eppendorf tubes 1 and 2 (Fig. 1B, step1), as mentioned in Materials and Methods. The BMBs from tubes 1 and 2 were pooled and distributed in the desired wells of the 96-well plate (Fig. 1B, step 1).

**(ii) Step 2: nucleic acid extraction and amplification of the pathogen target.** The test samples were processed for nucleic acid extraction, and pathogen targets were amplified by PCR (step 2, Fig. 1B) using two sets of primers targeted to major surface protein and flagellin, as mentioned in Materials and Methods. The reverse primer in each primer set was biotinylated, generating the biotinylated PCR products.

**(iii) Step 3: hybridization, fluorescence detection, and barcode decoding.** The biotinylated PCR product from each test sample was added to a specific well of a 96-well plate to allow the annealing of the biotinylated DNA strand of the PCR product with the cDNA probe tethered to the BMB (see hybridization protocol description in Materials and Methods). Unbound PCR products were washed. The biotinylated targets annealed to the DNA probe were identified using streptavidin, R-phycoerythrin conjugate (SAPE), a covalent conjugate of streptavidin protein, and a fluorescent label (phycoerythrin). The beads in each well were examined using the barcode detection system (BioCode 2500 Analyzer), which has a charged-coupled device (CCD) camera that takes the bright-field and fluorescence pictures of each well. The pictures were overlapped, and barcodes were scanned with the simultaneous measurement of the associated fluorescence signal. The positive fluorescence readout generated by a particular barcoded bead indicates the presence of pathogen in the test sample whose identity was revealed by decoding the barcode. A minimum of 25 BMBs per target per well are required for proper detection.

### Selection of targets and optimization of singleplex PCR.

**FIG 2:**
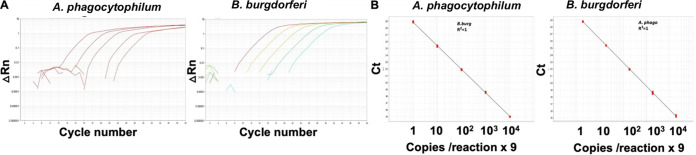
(A) Real-time PCR analysis of 10-fold dilution series of two synthetic gene blocks. (B) A plot showing the linear amplification of the targets using 10-fold dilution series of the gene block along with respective primer sets shown in Table 1.

Detection of the DNA genome in the test sample by the PCR approach is currently used for the diagnosis of A. phagocytophilum and B. burgdorferi. Selection of the highly conserved gene and selective amplification of the target region by the PCR is thus instrumental in correct diagnosis. Major surface protein (GeneID 56369034) is expressed on the outer membrane of A. phagocytophilum and is involved in host-pathogen interactions (32). This gene is highly conserved and has been used as a preferred target for the detection of A. phagocytophilum by the singleplex PCR-based methods (33, 34). Flagellin, a 41-kDa protein encoded by the highly conserved gene *flaB* (GeneID 56568071), is a major component of the periplasmic flagellar filament core of B. burgdorferi (35, 36). This gene has also been used in the diagnosis of B. burgdorferi by PCR-based methods (37). These two genes were subjected to BLAST searches against the genomes of 500 to 1,000 species of A. phagocytophilum and B. burgdorferi to identify the highly conserved regions in each gene. The highly conserved regions of ~200 nucleotides of the major surface protein and flagellin were synthesized by Integrated DNA Technologies (IDT), referred to here as gene blocks. Multiple probes and primer sets targeted to the gene block were similarly evaluated for cross-reactivity against the human, cattle, and canine genomes and the genomes of 500 to 1,000 species of other blood-borne pathogens. Finally, three probes and three primer sets targeted to the middle of the gene blocks, showing no *in silico* cross-reactivity, were tested for linear dynamic range and efficiency by SYBR green singleplex real-time PCR approach, using a 10-fold dilution series of the synthetic gene blocks (see Materials and Methods for details). As shown in Fig. 2, one of the primer sets (Table 1) showed linear amplification of the target with high sensitivity, detecting up to 9 copies of the gene block per test reaction for A. phagocytophilum *and* B. burgdorferi. The assay demonstrated 100% efficiency (*R*^2^ > 0.99) for the tested serial dilutions of each target. These primer sets (Table 1) were used for further development of the BMB assay.

**TABLE 1:** Primers and probes for A. phagocytophilum and B. burgdorferi used in the real-time PCR and BMB assays

Primer or probe	Anaplasma phagocytophilum	Borrelia burgdorferi
Gene target	Major surface protein	Flagellin
GeneID	56369034	56568071
Source/catalog no.	BEI/NR-50142	ATCC/35211
Forward primer	5′-TCCTGATCCTCGGATAGGGTT-3′	5′-TGAAATAGAGCAACTTACAGACGA-3′
Reverse primer	5′-Biosg/CCTGGCACCACCAATACCATA-3′′	5′-Biosg/TGTTCTTACATTTTGGGAAGCAGA-3′
Probe	5′-AmMC6/GGAAGGCAGTGTTGGTTATGGTATTGGTGG-3′	5′-AmMC6/TGCTGATCAAGCTCAATATAACCA-3′
Forward primer conc.	150 nM	150 nM
Reverse primer conc.	200 nM	200 nM

### Testing the detection of PCR amplicons using the BMB technology (singleplex).

**FIG 3:**
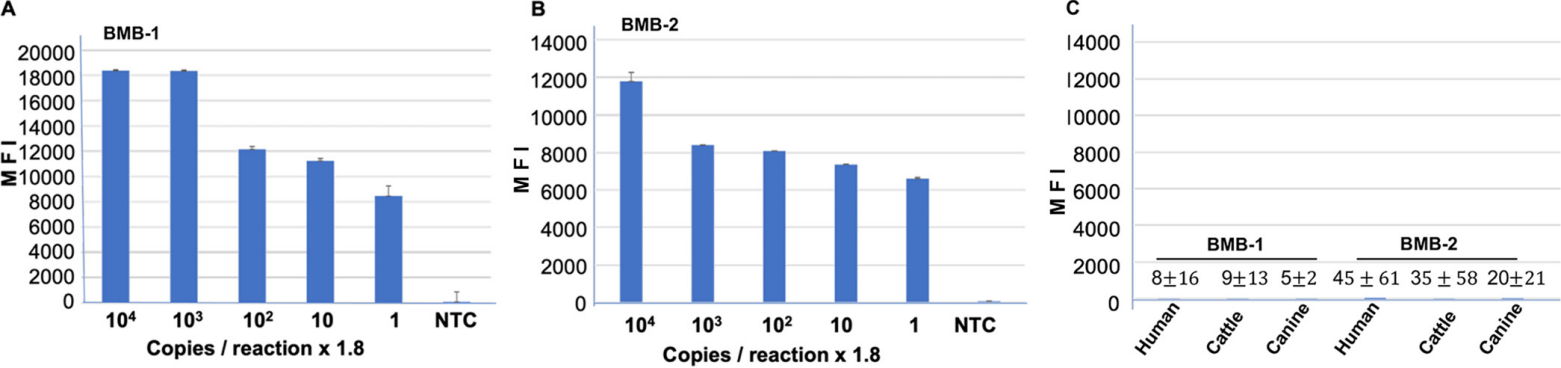
Detection of PCR amplicons using BMB technology. (A and B) Biotinylated PCR products obtained from 10-fold dilution series of synthetic gene blocks of the major surface protein of A. phagocytophilum (A) and the flagellin gene of B. burgdorferi (B) were detected by BMB technology. No-template control (NTC) represents the PCR during which no DNA template was added. (C) The biotinylated PCR products generated from DNA samples of human, cattle, and canine origin were detected using the BMB technology to show the background signal.

Probes complementary to the amplified regions of the major surface protein and flagellin (Table 1) were tethered to the barcoded magnetic beads (BMBs) with distinct barcodes referred as BMB-1 and BMB-2, respectively. The mixture of tethered BMB-1 and BMB-2 was distributed in a 96-well plate, as mentioned in Materials and Methods. PCRs in 25 μL were carried out using the dilution series of synthetic gene blocks along with the desired primer sets (Table 1), containing the biotinylated reverse primer. Then, 5 μL of the resulting biotinylated PCR products was incubated with the BMBs to allow the annealing of the PCR products with the complementary probe tethered to the BMBs, as mentioned in Materials and Methods. After annealing, the BMBs were washed to remove the unbound biotinylated PCR product, followed by further processing as mentioned in the assay workflow (Fig. 1, step 1). The 96-well plates were scanned, the BMBs showing the positive fluorescence readout were detected with the BioCode 2500 multiplex detection system, and the fluorescence intensity was recorded for the calculation of median fluorescence signal (MFI). The MFI was plotted against the number of copies of the gene block used in the PCR to demonstrate the sensitivity. As shown in Fig. 3, the BMB assay detected even two copies of the gene block in the PCR. The limit of detection (LOD) was later found to be 1.8 copies/reaction. It must be noted that number of probes attached to BMBs cannot be controlled; thus, the MFI signal does not linearly increase based on the number of copies of the gene block in the test reaction (Fig. 3). It is evident from Fig. 3 that the no-template control (NTC), a product from PCR lacking the DNA template, generated extremely weak MFI signal, demonstrating a significantly high signal to background ratio.

### Determination of cutoff value for MFI.

It is critical to determine the cutoff value for the output signal of a diagnostic assay to ensure the false-negative and false-positive samples are eliminated from the test results. Since our diagnostic assay tests the presence of A. phagocytophilum and B. burgdorferi in the test blood by quantifying the fluorescence readout of BMBs of interest, it is necessary to quantify the median fluorescence intensity (MFI) generated from test blood samples lacking the pathogen of interest to calculate the cutoff values for the MFI. We extracted the DNA from randomly selected blood samples of human, cattle, and canine origin and carried out the PCRs using the primer sets targeted to major surface protein of A. phagocytophilum and flagellin of B. burgdorferi (Table 1). The resulting biotinylated PCR products were hybridized with BMB-1 and BMB-2, and the resulting MFI signal was quantified, consistent with the assay workflow and plotted in Fig. 3C. The MFI signal in BMB-1 and BMB-2 from all samples from human (*n* = 14), cattle (*n* = 16), and canine blood (*n* = 14) was averaged, and the resulting MFI was found to be 8 ± 45 and 35 ± 51 for BMB-1 and BMB-2, respectively, which is negligible compared to the MFI signal generated in positive samples (compare Fig. 3A and B with Fig. 3C). The averaged MFI plus three standard deviations, which is equal to 143 and 188, represents the cutoff value for A. phagocytophilum and B. burgdorferi, respectively, in this duplex assay. However, it must be noted that MFI values for positive samples containing even two copies of the template were in multiples of thousands, generating a high signal to background ratio. As demonstrated later in the study, the huge difference in MFI signal between positive and negative samples dramatically reduces the false-positive results in this assay.

### Detection of A. phagocytophilum and B. burgdorferi by multiplex BMB technology.

Since multiplex PCRs contain numerous sets of primers targeted to different test pathogens, it is very important to determine that primer sets do not interfere in each other’s specificity and sensitivity during PCR amplification. In addition, the PCR amplicons must specifically anneal to their respective probes tethered to the BMBs at the hybridization step. To test this, we tethered the probe specific to A. phagocytophilum with the BMBs of a unique barcode, referred as BMB-1 (Tables 1 and 2). Similarly, the probe for B. burgdorferi was tethered to the BMB with a different barcode, referred as BMB-2, as mentioned above. For control, a random DNA probe was tethered to another BMB with a different barcode, referred as BMB-0. The barcodes were noted, and BMBs were mixed and distributed in different wells of the 96-well plate as mentioned in step 1 of the assay workflow (Fig. 1). Titered A. phagocytophilum obtained from ATCC was serially diluted from 2.8 × 10^5^ 50% tissue culture infection dose (TCID_50_)/mL to 2.8 TCID_50_/mL. Total DNA was extracted from 200 μL of the resulting dilution series in 50 μL of the elution buffer, as mentioned in Materials and Methods. Then, 5 μL of the resulting extracted DNA was used in a 25- μL PCR containing two sets of primers (one set of primers each for A. phagocytophilum and B. burgdorferi, shown in Table 1). Again, the reverse primer from each primer set was biotinylated, consistent with step 2 of the assay workflow. Then, 5 μL of the resulting biotinylated PCR product was added to the BMB mixture previously distributed in 96-well plates, to allow the hybridization between the tethered probe and the biotinylated PCR product. The beads were washed and processed, consistent with step 3 of the assay workflow. The MFI signal of BMB-0, BMB-1, and BMB-2 was recorded in each test well of the 96-well plate and reported in Table 2. It is clear from Table 2 that only BMB-1, harboring the probe specific to the target (A. phagocytophilum), showed a dramatic increase in MFI signal. The MFI signal of BMB-0 and BMB-2 was low and similar to the background with no template (NTC). The assay showed high sensitivity by detecting up to 2.8 TCID_50_/mL of A. phagocytophilum (Table 2). The LOD was also later found to be to 2.8 TCID_50_/mL. This clearly demonstrates that mixing primer sets does not interfere with sensitivity and specificity. The experiment was repeated exactly using the dilution series of the gene block, and the data are shown in Table 2. Again, the assay specifically detected 1.8 copies of the gene block per PCR (Table 2). The signal of BMB-0 and BMB-2 was again similar to the NTC background. Comparing the data from Table 2 with Fig. 3, mixing the primers did not impact sensitivity.

**TABLE 2:** Detection of A. phagocytophilum (Webster strain) by duplex BMB assay using the 10-fold dilution series of the titered pathogen^*a*^

TCID_50_/mL or copies/reaction^*b*^	MFI result^*b*^		
	BMB-0^*c*^	BMB-1^*c*^	BMB-2^*c*^
TCID_50_/mL			
2.8 × 10^5^	5 ± 3	23,131 ± 820	12 ± 9
2.8 × 10^5^	5 ± 4	18,647 ± 134	5 ± 3
2.8 × 10^5^	2 ± 0	22,714 ± 18	34 ± 32
2.8 × 10^5^	2 ± 1	14,563 ± 943	2 ± 1
2.8 × 10^5^	1 ± 0	21,564 ± 910	30 ± 28
2.8	3 ± 2	16,452 ± 750	3 ± 2
NTC	5 ± 0	15 ± 0	5 ± 0
Copies/reaction^*d*^			
1.8 × 10^5^	3 ± 1	24,835 ± 337	20 ± 16
1.8 × 10^4^	6 ± 4	18,520 ± 69	35 ± 26
1.8 × 10^3^	4 ± 0	16,337 ± 176	31 ± 14
1.8 × 10^2^	2 ± 1	12,325 ± 251	7 ± 5
1.8 × 10^1^	4 ± 1	8,825 ± 248	8 ± 2
1.8	8 ± 1	8,510 ± 293	11 ± 3
NTC	5 ± 0	15 ± 0	5 ± 0

a The titered pathogen A. phagocytophilum (TCID_50_/mL) was obtained from BEI (cat. no. NR-50142) to prepare the dilution series used in Table 2.

b NTC, no-template control.

c Barcoded magnetic beads BMB-0, BMB-1 & BMB-2 were tethered with the probes for the blank, A. phagocytophilum and B. burgdorferi, respectively.

d The gene block used in this table was synthesized by IDT, as mentioned in Materials and Methods.

Next, the titered B. burgdorferi from ATCC was similarly serially diluted from 1 × 10^3^ CFU/mL to 1 CFU/mL, and the resulting dilution series was similarly tested for sensitivity and specificity (Table 3). Again, the BMB-2 harboring the probe specific to B. burgdorferi showed a dramatic increase in MFI signal. The signal of BMB-0 and BMB-1 was similar to the NTC background. Similar results were obtained with dilution series of extracted genomic DNA of B. burgdorferi obtained from ATCC (Table 3). The assay showed high sensitivity by detecting 1 CFU/mL of titered pathogen and 4.5 copies of the B. burgdorferi genome per PCR. The LOD for B. burgdorferi was later found to be 1 CFU/mL.

**TABLE 3:** Detection of B. burgdorferi by duplex BMB assay using the 10-fold dilution series of the titered pathogen (B. burgdorferi) and the extracted genomic DNA of the pathogen (B. burgdorferi B31 strain)^*a*^

CFU/mL or copies/reaction^*a*^	MFI result^*c*^		
	BMB-0	BMB-1	BMB-2
CFU/mL			
1 × 10^3^	6 ± 2	32 ± 25	15,787 ± 750
1 × 10^2^	5 ± 1	43 ± 32	6,760 ± 214
1	4 ± 4	50 ± 32	4,467 ± 340
NTC^*b*^	1 ± 0	1 ± 0	1 ± 0
Copies/reaction^*d*^			
4.5 × 10^2^	3 ± 3	31 ± 20	9524 ± 368
4.5 × 10^1^	3 ± 3	6 ± 7	2822 ± 105
4.5	4 ± 4	16 ± 20	1159 ± 43
NTC^*b*^	2 ± 0	2 ± 0	2 ± 0

a The titered B. burgdorferi pathogen (CFU/mL) was obtained from ATCC (cat. no 35210) to prepare the dilution series.

b NTC, no-template control.

c Barcoded magnetic beads BMB-0, BMB-1, and BMB-2 were tethered with the probes for the blank, A. phagocytophilum, and B. burgdorferi, respectively.

d The quantified genomic DNA for *B. burgdorferi* used in dilution series was obtained from ATCC (Cat# 35210DQ).

### Simultaneous detection of both A. phagocytophilum and B. burgdorferi using the multiplex BMB technology.

We next confirmed that the sensitivity of the assay was retained when both the pathogens were present in the same sample. The high concentration-titered stocks of B. burgdorferi and A. phagocytophilum were mixed and serially diluted from 100 CFU/mL to 1 CFU/mL and 100 TCID_50_/mL to 1 TCID_50_/mL, respectively. Total DNA was extracted from 200 μL of the resulting dilution series, followed by PCR amplification using primer sets for both A. phagocytophilum and B. burgdorferi (Table 1). The PCR products were incubated with the mixture of BMB-0, BMB-1, and BMB-2 to allow annealing with the respective tethered probes, followed by detection as mentioned above. It is clear from Table 4 that both BMB-1 and BMB-2 showed a dramatic increase in the MFI signal consistent with the presence of both the targets in the test sample. Comparing Table 4 with Tables 2 and 3, it is evident that the sensitivity was retained in the simultaneous detection of both of the pathogens. The simultaneous detection was also confirmed using the dilution series of gene blocks (Table 4). Again, it is evident from Table 4 that unlike BMB-0, BMB-1 and BMB-2 showed the huge MFI signal, confirming the simultaneous detection of both of the pathogens. The MFI signal of BMB-0 was similar to that of the BMB-0 NTC background.

**TABLE 4:** Simultaneous detection of B. burgdorferi and A. phagocytophilum by duplex BMB assay using 10-fold dilution series of the pathogen and gene blocks^*a*^

Measurement	MFI result^*d*^		
	BMB-0	BMB-1	BMB-2
CFU or TCID_50_/mL^*b*^^,^^*c*^			
100	6 ± 1	10,902 ± 404	7,012 ± 266
10	9 ± 0	8,308 ± 315	4,871 ± 185
1	8 ± 0	4,724 ± 179	3,930 ± 143
NTC	9 ± 0	64 ± 0	23 ± 0
Copies/reaction/pathogen^*e*^			
1.8 × 10^3^	7 ± 1	16,484 ± 626	8,261 ± 313
1.8 × 10^2^	2 ± 0	14,113 ± 536	8,209 ± 311
1.8 × 10^1^	4 ± 0	10,523 ± 398	7,270 ± 276
1.8	5 ± 1	1,008 ± 381	6,413 ± 243
NTC	4 ± 0	4 ± 0	65 ± 0

a The gene block used was synthesized by IDT, as mentioned in Materials and Methods.

b CFU/mL is used for B. burgdorferi and TCID_50_/mL is used for (A. phagocytophilum).

c NTC, no template control.

d Barcoded magnetic beads BMB-0, BMB-1, and BMB-2 were tethered with the probes for the blank, A. phagocytophilum, and B. burgdorferi, respectively.

e A standard deviation of around 4% was added to each MFI value.

### Limit of detection (LOD).

Conservatively, guidance documents from the Food and Drug Administration (FDA) determine LOD as the minimum concentration of target analyte that is positively detected in 95% of specimen replicates tested. We observed that a test reaction containing no template (NTC) or extracted DNA from negative blood samples of human, cattle, or canine origin showed an MFI signal of less than 100. The test reaction containing even two copies of the target showed an MFI signal in multiples of 1,000 (Tables 2, 3, and 5). The high signal to background ratio is due the binding of many probes to a single bead and the use high-quantum-yield fluorophore in the final detection. This enables the BMB assays to detect even a single copy of the target in the test reaction, as reported in our FDA-approved assays (31). For LOD, we tested 10 replicates of A. phagocytophilum and B. burgdorferi as well their gene blocks at three concentrations (Table 6). It is evident from Table 7 that the observed LOD for A. phagocytophilum and B. burgdorferi is 2.81 TCID_50_/mL and 1 CFU/mL, respectively. The observed LOD for gene blocks is 1.8 copies/reaction for both the pathogens.

**TABLE 5:** Analytical specificity of the BMB assay using human blood spiked with blood-borne pathogens (10^5^ CFU/mL)

Sample name	No. of samples tested	MFI result^*a*^			
		BMB-1		BMB-2	
Babesia bovis	4	3 ± 2	Negative	27 ± 29	Negative
Babesia bigemina	4	4 ± 3	Negative	4 ± 3	Negative
Babesia microti	4	4 ± 2	Negative	4 ± 2	Negative
Ehrlichia canis	4	21 ± 33	Negative	11 ± 15	Negative
Ehrlichia chaffeensis	4	27 ± 44	Negative	31 ± 39	Negative
Ehrlichia ewingii	4	38 ± 37	Negative	30 ± 17	Negative
Human blood (not spiked)	14	8 ± 16	Negative	45 ± 61	Negative
Canine blood (not spiked)	14	5 ± 2	Negative	20 ± 21	Negative
Cattle blood (not spiked)	16	9 ± 13	Negative	35 ± 58	Negative

a Barcoded magnetic beads BMB-1 and BMB-2 were tethered with the probes for A. phagocytophilum and B. burgdorferi, respectively.

**TABLE 6:** LOD for the detection of *A. phagocytophilum* using strain Webster (BEI NR-5014) and synthetic gene blocks^*a*^

TCID_50_/ml	Positive/total tested		Gene block	Positive/total tested	
	BMB-1	BMB-2	Copies/reaction	BMB-1	BMB-2
2.81 × 10^1^	10/10	0/10	1.8 × 10^1^	10/10	0/10
2.81	10/10	0/10	1.8	10/10	0/10
0.281	03/10	0/10	0.18	03/10	0/10

a Barcoded magnetic beads BMB-1& BMB-2 were tethered with the probes for the detection of *B. burgdorferi A. phagocytophilum* and *B. burgdorferi*, respectively.

**TABLE 7:** LOD for the detection of *B. burgdorferi* using strain B-31 (ATCC 35210) and synthetic gene blocks^*a*^

CFU/ml	positive/total tested		Gene block	positive/total tested	
	BMB-1	BMB-2	Copies/reaction	BMB-1	BMB-2
1 × 10^1^	0/10	10/10	1.8 ×10^1^	10/10	0/10
1	0/10	10/10	1.8	10/10	0/10
0.1	0/10	4/10	0.18	03/10	0/10

a Barcoded magnetic beads BMB-1 and BMB-2 were tethered with the probes for the detection of *A. phagocytophilum* and *B. burgdorferi,* respectively.

### Performance evaluation using other blood-borne pathogens.

To ensure that probes and primer pairs do not cross-react with the host genome or with the genomes of other blood-borne pathogens and generate the false-positive results, the primer and probe sequences were evaluated for cross-reactivity using *In silico* analysis, as previously mentioned. This analysis revealed that selected primer pairs and probes (Table 1) show no cross-reactivity against the tested targets. To confirm this *in silico* observation, their specificity was evaluated against six other blood-borne pathogens, obtained from ATCC in high-titered stocks (Table 5). Total DNA was extracted from 200 μL of the human blood spiked with pathogens shown in Table 5 at the highest concentration (10^5^ CFU/mL), followed by detection using the BMB assay consistently with the assay workflow as mentioned above. Four samples for each pathogen at similar high concentrations were tested, and 100% of the tested samples showed the MFI value below the cutoff range, demonstrating the negative result (Table 5). Similar analysis was carried out with 10 blood samples each of human, cattle, and canine origin without the exogenous addition of any pathogen. These blood samples also showed negative results, further confirming the specificity of the developed BMB assay.

### Evaluation of diagnostic performance using spiked samples.

To further validate the performance of the diagnostic assay, we generated 20 spiked samples by exogenous addition of A. phagocytophilum to 20 randomly collected cattle blood samples, provided by Maisie Dawes, at a concentration of 2.81 TCID_50_/mL (1 × LOD). DNA was extracted from 200 μL of all spiked blood samples, as mentioned in Materials and Methods. For the negative control, DNA was also extracted from 10 cattle blood samples prior to the addition of A. phagocytophilum. The multiplex BMB assay detected all 20 A. phagocytophilum-positive samples (100% positive percent agreement), whereas all 10 nonspiked cattle blood samples tested negative (100% negative percent agreement). In addition, three no-template control (NTC) samples also tested negative, as expected (Table 8) . A similar strategy was used to examine the performance of the assay using the spiked B. burgdorferi samples at a concentration of 1× LOD. Again, 100% positive and negative agreements were observed (Table 8).

**TABLE 8:** Performance of the BMB assay using the spiked blood samples^*a*^

Sample name	No. of samples	MFI ± SD		
		BMB-1^*c*^	BMB-2^*c*^	Result
NTC-not spiked^*b*^	3	7 ± 1	4 ± 1	Negative
Cattle blood-not spiked	10	9 ± 13	35 ± 58	Negative
Cattle blood spiked (*Anaplasma)*	20	6,024 ± 1973	5 ± 1	Positive
Cattle blood spiked (*Borrelia)*	20	*1*7 ± 1	3,792 ± 1,007	Positive

a Cattle blood was spiked with 1 × LOD of either A. phagocytophilum or B. burgdorferi.

b NTC, no template control.

c Barcoded magnetic beads BMB-1 and BMB-2 were tethered with the probes for the detection of A. phagocytophilum and B. burgdorferi, respectively. The values for cattle blood not spiked were also reported in Table 5.

### Evaluation of diagnostic performance using patient samples.

**FIG 4:**
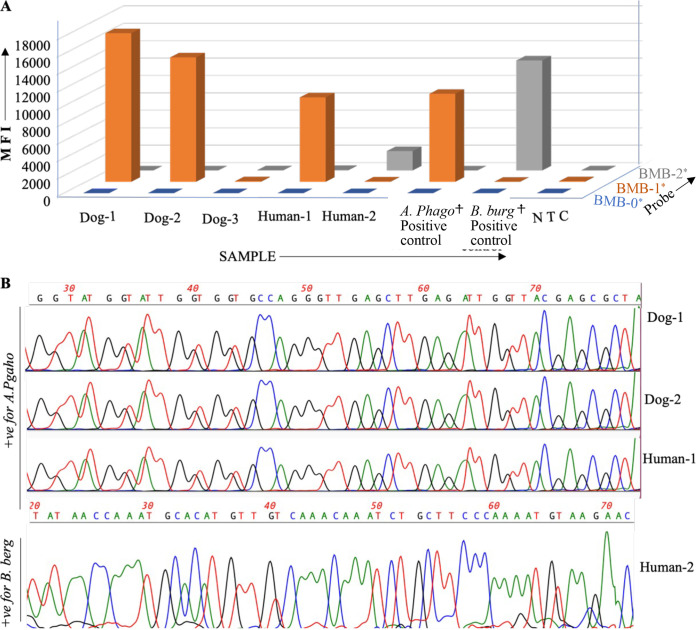
Evaluation of diagnostic performance using patient samples. (A) Test results of patient samples. (B) A representative region of DNA sequence obtained by sequencing the extracted DNA from patient samples. The source of the patient samples is mentioned in the manuscript text.

Since the duplex BMB assay showed good sensitivity and specificity, we next wanted to determine whether it can detect the pathogens in the patient samples. We received several dog and human samples from Tonatiuh Melgarejo, a colleague and veterinarian here at the College of Veterinary Medicine, Western University of Health Sciences. Based on the history and symptoms, the clinician suspected A. phagocytophilum infection in dogs. Blood samples were obtained from three suspected dogs and two suspected humans. Total DNA was extracted from 200 μL of the blood. For the positive control, titered A. phagocytophilum from BEI Resources and B. burgdorferi from ATCC were diluted in the negative human blood, followed by DNA extraction. The extracted DNA was PCR amplified using primer sets for both A. phagocytophilum and B. burgdorferi (Table 1), followed by detection using the assay workflow, as mentioned in Fig. 1. Analysis of the data revealed A. phagocytophilum infection in one human and two dog samples (Fig. 4). The second human sample tested positive for B. burgdorferi, and the third dog sample was negative for both pathogens. The results were confirmed by sequencing the extracted DNA samples from blood samples (Fig. 4). The representative sequencing data (Fig. 4B) show 100% sequence homology with the respective pathogens.

## DISCUSSION

Due to global warming (1), the rising tick population has expanded to areas where they were previously unable to survive (38). In the United States, ticks are responsible for over 95% of vector-borne diseases (39). According to these reports (24, 39) LD is the most prevalent tick-borne illness in the Northern Hemisphere and causes approximately 300,000 infections annually in the United States alone. However, only 10% of tick-borne infections are reported to the CDC (40, 41). Based on 42,743 cases of LD reported to the CDC in 2017, the annual cost is estimated to be over 500 million dollars in the United States alone (40) and over tens of millions of euros in European countries (42). The posttreatment LD syndrome (43), disability benefits (44), and loss of tourism (45) would further increase these costs. Tick-borne diseases (TBD) inflict a loss of 18.7 billion U.S. dollars to the cattle industry worldwide (46). In Tanzania alone, an estimated mortality of 1.3 million cattle by TBD causes an annual economic loss of 364 million U.S. dollars (47). These losses to human and animal life are mostly due to the lack of efficient, cost-effective, sensitive, and modern diagnostic approaches for TBD. There is no FDA-approved multiplex diagnostic approach for rapid, accurate diagnosis and surveillance studies for TBD (48). The existing serology-based singleplex diagnostic assays for A. phagocytophilum and B. burgdorferi (49, 50) and other TBD (51) are costly, lack sensitivity, and cannot be used in high-throughput mode to gauge prior insight about the prevalence of TBD in a geographical area. Due to the low pathogen load in test blood samples, the detection of B. burgdorferi by conventional PCR methods has been highly inefficient. However, prior culturing of B. burgdorferi under laboratory conditions followed by PCR has resulted in better detection rates (52). Advances in DNA extraction methods and amplification techniques have improved the detection of low copy numbers of *Borrelia* DNA from larger patient sample volumes (53). Newer PCR techniques, such as real-time quantitative PCR (qPCR) and nested PCR, have demonstrated improved sensitivity for the detection of B. burgdorferi (52, 54). A digital PCR method has been reported to detect up to 3 copies of B. burgdorferi genome in the test sample (14). The real-time PCR-based assays can detect 5 to 10 copies of A. phagocytophilum target DNA in the test sample (23). However, to our knowledge, none of these singleplex PCR-based methods are approved by the FDA for clinical diagnosis of LD and anaplasmosis.

The BMB assay developed in this study demonstrates the proof of the principle for the simultaneous detection of A. phagocytophilum and B. burgdorferi from a single patient sample. Using additional probes and primer sets specifically targeted to other tick-borne pathogens, the assay can be upgraded to a high-throughput multiplex diagnostic approach for simultaneous detection of all clinically important tick-borne pathogens from a single patient sample. Such a multiplex diagnostic approach will be instrumental in surveillance studies of tick-borne diseases and routine clinical testing in diagnostic centers.

In BMB technology numerous probes complementary to the target amplicon are attached to a single bead, which facilitates the binding of numerous complementary biotinylated DNA strands from PCR-amplified targets during the hybridization step to each BMB. In addition, the fluorophore with high quantum yield is used in the final detection. Binding of numerous probes along with the use of high-quantum-yield fluorophore significantly increases the sensitivity of this diagnostic approach. This is evident from the detection of ~2 copies of the gene block in each test reaction with the BMB assay in comparison to 9 copies of gene blocks per reaction with the SYBR green real-time PCR assay (compare Fig. 2 with Fig. 3 and Table 6). Using titered pathogens, the LODs for the detection of B. burgdorferi and A. phagocytophilum were 1 PFU/mL and 2.8 TCID_50_/mL, respectively (Table 6). Since the number of probes bound per BMB cannot be controlled, the MFI signal does not linearly increase by increasing the concentration of the target pathogen in the test reaction (Tables 2 to 4). Nonetheless, the assay has an extremely high signal to background ratio, evident from negligible MFI signal in the NTC (Fig. 3, Tables 2 to 4) and blood samples lacking the exogenously added A. phagocytophilum or B. burgdorferi (Fig. 3C and Table 8). The MFI signal in positive samples containing even 2 copies of the gene block was several thousand-fold higher than the MFI signal from the NTC (Tables 2 to 4). The high signal to background ratio helps in the elimination of false-positive and false-negative test results and thus makes the diagnostic results more reliable. The assay has efficient reaction kinetics favored by the mixing of BMBs with the test samples in liquid suspension. The assay is currently run in 96-well plates but can be upgraded to 384-well plates, which will be instrumental in surveillance studies to gauge prior insight about the prevalence of A. phagocytophilum and B. burgdorferi in a geographical area. This will ultimately help in preventing a potential bigger outbreak. The performance studies revealed high specificity and sensitivity, evident from the lack of cross-reactivity with other blood-borne pathogens or with the genomic DNA extracted from blood samples of human, cattle, or canine origin. The performance studies using spiked pathogens revealed 100% positive and negative agreement (Table 8). The assay was selectively able to detect the test pathogens in the canine and human patient samples, which was further confirmed by the sequencing studies (Fig. 4). A brief cost analysis (not shown) demonstrated that a BMB multiplex diagnostic assay capable of detecting up to 18 tick-borne pathogens in a single patient sample will be of similar cost as a singleplex PCR assay that can detect a single pathogen in the test sample. Such cost-effectiveness is due to the low cost of BMBs and the use of only 25 BMBs per target. Taken together, our results demonstrate the proof of the principle for the simultaneous detection of A. phagocytophilum or B. burgdorferi in a test sample with a limit of detection of 2.81 TCID_50_/mL and 1 CFU/mL, respectively, using the BMB technology. The reported results demonstrate the proof of the principle that this duplex BMB assay can be upgraded to a high-throughput multiplex diagnostic approach for simultaneous detection of numerous clinically important tick-borne pathogens.

## MATERIALS AND METHODS

### Primer and probe design.

Sequences encoding the major surface protein (GeneID 56369034) and flagellin (GeneID 56568071) of A. phagocytophilum and B. burgdorferi, respectively, were downloaded from the National Center for Biotechnology Information (NCBI). Using BLAST (https://blast.ncbi.nlm.nih.gov/Blast.cgi) (55), 500 to 1,000 sequences of each gene were identified and downloaded. The resulting sequences were aligned using Jalview, Multiple Sequence Alignment Viewer (MSA), and Clustal Omega to identify highly conserved regions for PCR amplification to generate amplicons of 100 to 200 bp in length. Using BLASTN, the amplicons were evaluated for potential cross-reactivity with closely related organisms. A minimum of five probe and primer sets for each amplicon were designed using primer3 and MacVector. The primers with a length of 18 to 25 nucleotides and a GC content of 50% or higher were preferred for synthesis. The primer characteristics, including the melting temperature (*T_m_*), formation of hairpins, self-dimers, and heterodimers, were examined using the OligoAnalyzer tool from Integrated DNA Technologies (IDT, San Diego, CA). For the barcoded magnetic bead assay (BMB assay), the reverse primer contained the biotin modification, whereas the probe contained amino modifications at the 5′ terminus. A standard (cytosin)_6_ spacer arm was introduced between the primer sequence and the 5′ modified nucleotide. All primers and probes were synthesized by IDT.

### Samples: gene blocks, titered pathogens, and DNA from whole blood).

Gene fragments of ~200 nucleotides in length corresponding to highly conserved regions of major surface protein (A. phagocytophilum) and flagellin (B. burgdorferi), harboring the test amplicons of interest, were synthesized by IDT. A. phagocytophilum (Webster strain, catalog [cat.] no. NR-50142) and B. burgdorferi (strain B31, cat. no. 35210) were obtained from BEI and ATCC, respectively. DNAs extracted from the whole-blood human and dog samples were a gift from Tonatiuh Melgarejo, and cattle blood was a gift from Maisie Dawes, both faculty members at the College of Veterinary Medicine, Western University of Health Sciences. Leftover canine blood from Pet Hospital at Western University of Health Sciences was used in this study, consistent with the approved Institutional animal care and use committee (IACUC) protocol (R221ACUC009). Leftover human blood was obtained from a commercial vendor (All Cells, Inc., California) in accordance with the exempt status of the approved institutional review board (IRB) protocol (X22/IRB/031).

### Coupling of DNA probes with barcoded magnetic beads (BMBs).

BMBs with two types of barcodes were chemically linked with probes complementary to the gene encoding either the major surface protein or flagellin, using EDC [1-ethyl-3-(3-dimethylaminopropyl) carbodiimide], following a well-standardized hybridization protocol routinely used in our lab (31). Briefly, the EDC solution was freshly prepared by dissolving 10 mg of EDC in one mL of cold 50 mM morpholineethanesulfonic acid (MES) buffer (pH 5.0). Around 10,000 BMBs, stored in phosphate-buffered saline with Tween 20 (PBST), were washed twice with 200 μL of 50 mM MES with Tween 20 (MES-T) buffer (pH 5.0), followed by centrifugation at 1,200 rpm for 30 s. The supernatant was discarded, and the pelleted beads were incubated in a 1.5-mL microcentrifuge tube for 5 to 10 min with 1 μL of DNA probe of interest (200 nM stock) along with 40 μL of the freshly prepared EDC solution, with continuous shaking at 1,600 rpm on a BioShake XP instrument at room temperature for 2 h. The resulting mixture was centrifuged at 1,200 rpm for 30 s, and the tubes were placed on a magnetic stand for 20 to 30 s, followed by careful removal of the supernatant. Next, the pelleted BMBs were incubated with 1 mL of 50 mM Tris-HCl, pH 7.4, for 15 min at room temperature, with continuous shaking at 1,600 rpm. The mixture was centrifuged at 1,200 rpm, and the pelleted beads were washed twice with 500 μL of PBS. The pelleted beads were blocked with 500 μL of 1% bovine serum albumin (BSA) in PBS for 1 h at room temperature, with constant shaking at 1,600 rpm. The mixture was centrifuged at 1,200 rpm for 30 s at room temperature, and the pelleted beads were washed twice with 500 μL of PBST, pH 7.4, and finally resuspend in 500 μL of 8× saline sodium phosphate EDTA (SSPE) buffer, pH 7.4, for longer storage at 2 to 8°C. The 8× SSPE buffer was prepared by diluting a 20× stock, which contains 3.0 M NaCl, 0.2 M NaH_2_PO_4_, and 0.02 M EDTA at pH 7.4. Antimicrobial reagent (0.1% Proclin-950) was added to the 20× SSPE stock buffer for longer storage. This coupling reaction can be scaled up for coupling large quantities of BMBs with respective probes and stored at 2 to 8°C for later use.

### DNA extraction and PCR amplification of the target gene.

Total DNA was extracted from 200 μL of the test sample using the nucleic acid extraction kit (Qiagen, cat. no. 56304), following the manufacturer’s instructions. The test samples at different concentrations were generated by diluting the target pathogens (A. phagocytophilum and B. burgdorferi) from high-concentration stock in universal transport medium or whole blood. Total DNA was eluted in 50 μL of Tris-EDTA (TE) elution buffer and stored at 2 to 8°C for next day use or stored at −80°C for long-term storage. Before carrying out the PCR amplification of the target gene, large volumes of PCR master mix (500 μL) were prepared in a 1-mL Eppendorf tube by the addition of 250 μL of 2× polymerase master mix (Promega, cat. no. D6006), forward primer (150 nM), reverse primer (200 nM), and nuclease-free water to a final volume of 500 μL. PCRs (25 μL) were assembled by adding 20 μL of the resulting PCR master mix in each well of a 96-well plate, followed by the addition of 5 μL of the extracted DNA template. The 96-well plates were sealed and briefly centrifuged to collect the samples at the bottom of the well. PCR amplification was carried out using a PCR cycler (Applied Biosystems, Veriti) with the following cycling conditions: single denaturation step at 94°C for 30 s, followed by 40 cycles of amplification with an annealing temperature of 58°C for 30 s and amplification of the target at 72°C for 30 s. PCR optimizations was done using asymmetric primer concentrations in the mix. Primer sequences and concentrations used in the final BMB assay are shown in Table 1. To avoid contamination, master mix preparation, DNA extraction, and PCR amplification were done in separate rooms.

### Hybridization.

The biotinylated PCR products were hybridized with the BMBs tethered to the DNA probes for the detection of A. phagocytophilum and B. burgdorferi. A total of ~50 to 100 BMBs were pipetted into a 5-mL tube from the BMB mix, which was generated by mixing the BMBs tethered to probes specific for major surface protein or flagellin (as mentioned in the BMB coupling section above) in equal ration. At least 25 BMBs coupled to a specific probe are required for appropriate detection of the target in each well of the 96-well plate. The 5-mL tube was then placed on the magnetic stand, and 8× SSPE buffer was carefully aspired, followed by the addition of 50 μL of hybridization buffer (50 mM Tris-HCl, pH 8.0) to the beads. The number of beads and the hybridization buffer can be scaled up depending on the number of samples to be tested. The BMBs in 5-mL tubes were mixed vigorously and pipetted up and down at least 5 times before 45 μL was dispensed in each well of the 96-well plate. Flat-bottom 96-well plates from Greiner Bio-One (part no. 655101) were used in this study. Biotinylated PCR product (5 μL) was added to the desired wells of the 96-well plate, followed by pipetting up and down three times. The plate was incubated for 10 to 30 min at 52°C with continuous shaking at 700 rpm (Labnet Vortemp 56). Next, the plate was placed on the magnetic microplate separator (Promega), and the supernatant was aspired. Then, 50 μL of the SAPE solution (2.5 μg of SAPE/mL in hybridization buffer), prepared by diluting the high-concentration original stock (Moss Bio. Inc., cat. no. SAPE-001), was added to each well, and the plate was incubated at 52°C for 5 to 15 min with continuous shaking at 700 rpm. Each well of the plate was washed twice with 500 μL of PBST buffer, pH 7.4, containing 0.1% Tween 20, by pipetting the contents up and down 3 times, followed by the removal of supernatant using a magnetic microplate separator or BioTek washer (ELx50 Model 8M or equivalent containing the magnet). Finally, 200 μL of PBST buffer was added to each well, and the contents were carefully pipetted up and down 10 times, and the formation of air bubbles was avoided. The plate was sealed with a clear/transparent plate cover and scanned with the BioCode 2500 BMB analyzer.

### Real-time PCR and gel electrophoresis of amplicons.

Real-time PCRs (25 μL) were assembled by adding 12.5 μL of 2× SYBR green master mix (Thermo Fisher, cat. no. A25742), 2.5 μL of forward primer (100 μM), 2.5 μL of reverse primer (100 μM), 5 μL of extracted DNA from the test sample, and 2.5 μL of water. Cycling conditions included the initial melting step of 95°C for 2 min, followed by 40 cycles consisting of incubations at 95°C for 30 s, 58°C for 30 s, and 72°C for 30 s, for each cycle. The samples were next incubated at 72°C for 2 min, followed by a long-term hold at 4°C. The threshold cycle (*C_T_*) values were calculated as previously reported (56, 57). Efficiency of PCR was determined using the linear regression equation (58). All PCRs were carried out in duplicates and repeated 2 to 3 times. Melting curves for each PCR were analyzed, and the PCR products were further examined using agarose gels.

### Sequencing of PCR products.

The PCR products from clinical samples were sent for sequencing (MCLAB, San Francisco, CA) for the confirmation of positive results obtained using BMB technology. Sequencing data were analyzed using MacVector.
